# Diet Alters Serum Metabolomic Profiling in the Mouse Model of Chronic Chagas Cardiomyopathy

**DOI:** 10.1155/2019/4956016

**Published:** 2019-12-20

**Authors:** Kezia Lizardo, Janeesh Plakkal Ayyappan, Usha Ganapathi, Walderez O. Dutra, Yunping Qiu, Louis M. Weiss, Jyothi F. Nagajyothi

**Affiliations:** ^1^Department of Microbiology, Biochemistry and Molecular Genetics, Public Health Research Institute, New Jersey Medical School, Newark, USA; ^2^Laboratory of Cell-Cell Interactions, Instituto de Ciências Biológicas, Departamento de Morfologia, Belo Horizonte, Brazil; ^3^Department of Medicine, Albert Einstein College of Medicine, New York, USA; ^4^Department of Pathology, Albert Einstein College of Medicine, New York, USA

## Abstract

Chagas disease is caused by *Trypanosoma cruzi* which is endemic in Latin America. *T. cruzi* infection results in a latent infection with approximately a third of latently infected patients developing chronic Chagas cardiomyopathy (CCM). CCM is a common cause of cardiomyopathy in endemic regions and has a poor prognosis compared to other cardiomyopathies. The factors responsible for the transition from the asymptomatic indeterminate latent stage of infection to CCM are poorly understood. Our previous studies demonstrated that lipid metabolism and diet are important determinants of disease progression. In the present study, we analyzed various serum metabolomic biomarkers such as acylcarnitines, amino acids, biogenic amines, glycerophospholipids, and sphingolipids in murine models of CCM, where the mice specifically develop either left or right ventricular cardiomyopathy based on the diets fed during the indeterminate stage in a murine model of Chagas disease. Our data provide new insights into the metabolic changes that may predispose patients to CCM and biomarkers that may help predict the risk of developing cardiomyopathy from *T. cruzi* infection. *Author Summary*. Chronic Chagas cardiomyopathy (CCM) is a parasitic disease prevalent in Latin America. Currently, no effective drugs or vaccines are available to prevent or cure CCM. The factors involved in the disease severity and progression are poorly understood to design new therapeutic interventions. In order to rapidly identify Chagas patients with a higher risk to develop CCM, a new set of biomarkers specific to Chagas disease is needed. We performed serum metabolomic analyses in chronic *T. cruzi*-infected mice fed on different diets and identified cardiac ventricular-specific metabolite biomarkers that could define CCM severity. In this paper, we present the results of serum metabolomic analyses and discuss its correlations to the diet-induced metabolic regulations in the pathogenesis of CCM in a murine model of Chagas disease.

## 1. Introduction

Chagas disease, caused by the protozoan parasite *Trypanosoma cruzi*, is endemic to Latin America, where approximately 8-10 million people are infected [1]. Following the acute phase of infection, most infected individuals enter into a prolonged asymptomatic form of disease termed the “chronic indeterminate phase,” which can persist for life without developing Chagas-related symptoms [2]; however, approximately 30% of “chronic indeterminate phase” individuals will develop debilitating and sometimes life-threatening Chagas-related symptoms including chronic Chagas cardiomyopathy (CCM) [3]. It is estimated that the number of annual deaths due to CCM is around 50,000. Of these, 60% are related to sudden cardiac death (SCD), 25% to heart failure, and 15% to stroke [4]. Chagas disease is a major cause of heart disease and cardiovascular-related deaths in Latin America.

Chronic Chagas cardiomyopathy is characterized by its various degrees of severity, and Chagas patients have a poorer prognosis than non-Chagas cardiac patients [5]. There are no vaccines or effective drugs to prevent or treat chronic Chagas cardiomyopathy. Furthermore, the lack of prognosis and progression markers for chronic Chagas disease is a barrier for testing new drugs to prevent the progression of cardiomyopathy. Several inflammatory and protein molecules such as NT-proBNP and Hs-cTnT have been identified as diagnostic biomarkers in distinguishing the severity of CCM [6, 7], but these markers are not specific to this cardiomyopathy [8]. There has been a lack of biomarker identification for Chagas disease severity-specific biomarkers that could predict the risk of developing cardiac dysfunction/cardiomyopathy during the asymptomatic indeterminate phase. Identification and development of such biomarkers would help to develop strategies to prevent the transition from indeterminate to symptomatic stage.

Our research has identified that *T. cruzi* infection results in cardiac lipidopathy [9]. *T. cruzi* binds to cholesterol-rich lipoproteins and invades host cells through LDL receptors and other scavenger receptors, resulting in intracellular lipid accumulation [10], and in CCM, there are significantly increased lipid levels in the myocardium [9]. Increased intracellular lipids impair lipid metabolism in the myocardium exacerbating mitochondrial oxidative and ER stress and exhaust mitochondrial oxidative capacity, which contributes to the development of CCM [11]. CCM, which develops after several years of infection, is essentially an immunometabolic disease.

Various mouse models of Chagas disease have been used to investigate cardiac pathology during acute and chronic stages of infection [12, 13]. Previously, we demonstrated that diet plays a major role in determining cardiac pathology in *T. cruzi*-infected CD1 mice [14, 15]. In particular, a high-fat diet (HFD) significantly modulates cardiac pathology and improves survival during acute infection as compared to mice fed on a regular diet (RD) that is carbohydrate-rich [14, 15]. HFD and RD contain the same protein calories and differ in fat and carbohydrate-derived calories. When the *T. cruzi*-infected mice were fed on either a HFD or a RD during the indeterminate stage, i.e., starting after the end of acute infection to late chronic stage (from 35 DPI to 150 DPI), they developed diet-specific ventricular enlargements during chronic infection [16]. These studies in a murine model of chronic Chagas disease established an interesting hypothesis that a long-term HFD treatment causes right ventricular dilation and wall thinning, and a long-term RD treatment results in left ventricular dilation [16]. This suggests that specific diets can alter the metabolic status in the host, which in turn regulates cardiac pathogenesis specific to either left ventricle (LV) or right ventricle (RV). Therefore, the abundance of specific serum metabolites in *T. cruzi*-infected mice fed on different diets during the indeterminate and early chronic phases may be indicative of disease progression specific to LV and RV.

In Chagas disease patients, dysfunction and dilation only of the LV have historically been studied; however, recent studies have demonstrated that RV dysfunction and dilation are predominantly seen in Chagas disease patients with heart failure [1, 17]. Enlargement of the liver and other signs of systemic and pulmonary congestion are also commonly observed in severe CCM patients who die after the onset of heart failure [18].

Alterations in the global metabolomic profiling during the acute stage of *T. cruzi* infection in a murine model have been demonstrated [19]. However, the metabolomic profiles differ between acute and chronic stages of Chagas disease, and the progression to CCM is associated with the chronic phase of infection. Herein, we report the first analysis of the serum metabolic profile in an experimental model of chronic Chagas disease and identify potential metabolite biomarkers that are associated with the progression from the indeterminate to the symptomatic stage of disease. We also demonstrate the effect of various diets on glucose tolerance and hepatic lipid metabolism at 150 days postinfection (DPI).

## 2. Materials and Methods

### 2.1. Mouse Infection and Sample Collection

A global metabolomic analysis of mice was used to assess the effect of diets on the pathogenesis of chronic Chagas cardiomyopathy and host metabolism. *T. cruzi* infection and maintenance of infected mice have been previously described [14, 16]. In brief, male 6-8 weeks CD1 mice (purchased from Jackson Laboratory) were infected intraperitoneally (i.p.) at 6 to 8 weeks of age with 5 × 10^3^ trypomastigotes of the Brazil strain and fed on rodent chow diet (PicoLab Mouse Diet 20 #5058 containing 23.19% calories of protein, 21.63% fat, and 55.17% carbohydrates). After 35 days postinfection (after acute infection, 35 DPI), mice were randomly divided into two groups (*n* = 20 per group) and fed on either a high-fat diet (HFD; 60% fat calories (20% calories of protein and 20% calories of carbohydrates) D12492 Research Diets, Inc., New Brunswick, NJ) or a low-fat control diet (RD; 10% calories of fat). Although RD diet was designed to be used as a control diet, it is also considered a carbohydrate-rich diet (RD; 70% carbohydrate calories (20% calories of protein and 10% calories of fat)) when compared to the “standard” rodent diet PicoLab #5058. [14, 16]. Uninfected mice were fed on either HFD (*n* = 20) or RD (*n* = 20) and used as respective controls in all the experiments. Mice were euthanized and livers were harvested after a cardiac MRI imaging analysis for biochemical analysis at 150 DPI [16]. Serum samples were obtained from 75 *μ*l of blood collected from the orbital venous sinus (using isoflurane anesthesia) 150 DPI. The experiment was repeated to confirm all results.

### 2.2. Ethics Statement

All animal experimental protocols were approved by the Institutional Animal Care and Use Committee (IACUC) of the Albert Einstein College of Medicine (No. 20130202) and the Rutgers Biomedical and Health Sciences (No. 15107), which adhere to the National Research Council guidelines (Guide for the Care and Use of Laboratory Animals: Eighth Edition, Washington, DC: The National Academies Press, 2011).

### 2.3. Fasting Blood Glucose

Tail blood was drawn seven hours after food was removed. Glucose was measured on whole blood with AlphaTRAK 2 blood glucose test strips and monitoring system (Abbott) [15].

### 2.4. Oral Glucose Tolerance Test (OGTT)

After a 6-hour fast, the mice (5 per group) were weighed and their base line glucose was measured using an AlphaTRAK® Blood Glucose Monitoring System. Mice were gavaged orally with a glucose solution of 2 mg glucose/g body weight, and blood glucose was measured after 15, 30, 60, and 120 minutes [15].

### 2.5. Sample Processing and Metabolomic Analysis

Serum samples were measured using the AbsoluteIDQ p180 targeted metabolomic kit (Biocrates Life Sciences AG, Innsbruck, Austria), and a UPLC-MS/MS (Xevo TQ, Waters, Pittsburgh, PA, USA) following the manufacturer's instruction. Serum samples were prepared according to the manufacturer's instructions adding several stable isotope-labelled standards to the samples prior to the derivatization and extraction steps. Using LC/MS, up to 184 metabolites from 5 different compound classes, namely, acylcarnitines, amino acids, biogenic amines, glycerophospholipids, and sphingolipids can be quantified [20]. Sample order was randomized, and pooled quality control (QC) samples (minimum 3) were plated at different positions on the 96-well plate and injected multiple times for covariance variation (CV) calculation for data quality control. Data were normalized between batches using the results of quality control level 2 repeats across the plate and between plates using Biocrates METIDQ software. Metabolites with <20% CVs were treated as accurate quantification, and CVs between 20 and 30% were treated as relatively accurate quantification. Metabolites with CVs > 30% were excluded.

Heat maps were used to depict the relatively altered and unbalanced metabolic signature among different groups of mice (infected RD-fed, uninfected HFD-fed, and infected HFD-fed mice) compared to uninfected RD mice. Heat maps were generated using the Excel software based on the abundance of the differentially expressed metabolite data with the increased metabolite levels colored in red and decreased metabolite levels colored in green compared to the level of specific metabolite in uninfected RD mice (colored in pale yellow). To identify diet-specific alterations in the left and right ventricle pathology, we compared the uninfected and the infected groups fed on the respective diets and a percentage change was calculated.

### 2.6. Immunoblot Analysis

Protein analysis was performed as previously described [14, 16] using the following antibodies: fatty acid synthase (FAS) Rabbit Polyclonal antibody (1 : 1000 dilution, ab22759) from Abcam Inc. (Cambridge, MA); phospho-ATP-citrate lyase (pACL) (Ser455) Rabbit Polyclonal Antibody (1 : 1000 dilution, #4331), Cell Signaling Technology; AceCS1 (D19C6) Rabbit monoclonal Ab (1 : 1000 dilution #36585), Cell Signaling Technology; Phospho-Acetyl-CoA Carboxylase (pACC) (Ser79) Rabbit Polyclonal Antibody (1 : 1000 dilution #3661), Cell Signaling Technology; anti-CPT1A, mouse monoclonal antibody (CPT1) 1 : 1000 dilution (8F6AE9) (ab128568), Abcam Inc.; Anti-Lipin 1 Rabbit Polyclonal antibody (Lipin 1) 1 : 1000 dilution (ab70138) Abcam Inc.; and Peroxisome Proliferator Activated Receptor Alpha Rabbit Polyclonal antibody (PPAR*α*) 1 : 1000 dilution (PA1-822A), ThermoFisher. Horseradish peroxidase- (HRP-) conjugated goat anti-mouse immunoglobulin (1 : 2000 dilution, Thermo Scientific Catalog #32230) or horseradish peroxidase- (HRP-) conjugated goat anti-rabbit immunoglobulin (1 : 2000 dilution, Thermo Scientific Catalog #31463) was used to detect specific protein bands (explained in the figure legends) using a chemiluminescence system [14]. GDI (Rabbit Polyclonal 1 : 10000 dilutions, Thermo Scientific QG223848) and a secondary antibody horseradish peroxidase-conjugated goat anti-rabbit (1 : 2000 dilution, Thermo Scientific Catalog #31463) were used to normalize protein loading [16].

### 2.7. Cholesterol Measurement

Cholesterol levels were quantified in the hearts and livers of mice at 150 DPI using a colorimetric assay kit, and samples were prepared and assayed following the manufacturer's protocol (total cholesterol colorimetric assay kit, Cell Biolabs Inc., CA).

### 2.8. Statistical Analysis

Statistically significant differences were tested using Student's *t*-test, and the corrected *p* values (*q*-value) < 0.05 were deemed as statistically significant. The identified metabolites influenced by *T. cruzi* infection and/or diet compared to uninfected RD mice are presented according to *p* values.

## 3. Results

### 3.1. Experimental *T. cruzi* Infection

**Figure 1:**
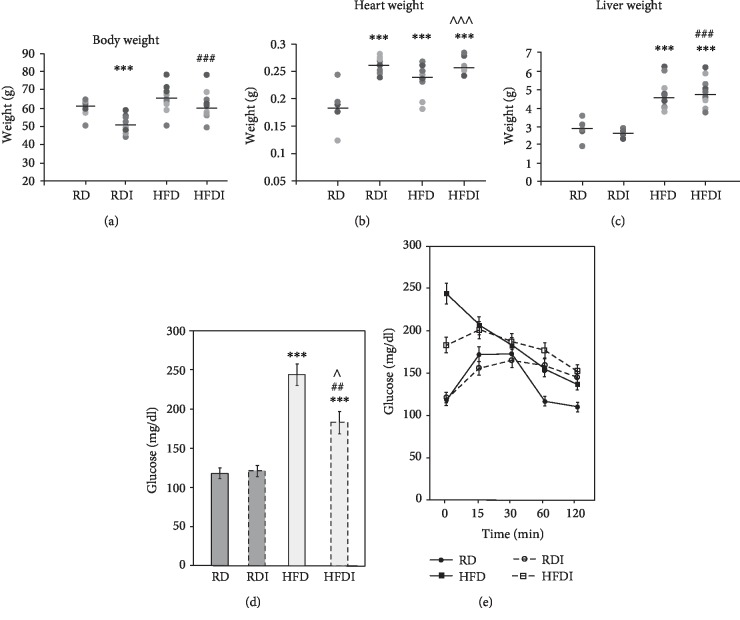
*T. cruzi* infection altered body weights and glucose levels in chronic mice fed on different diets. (a) *T. cruzi*-infected HFD mice displayed greater body weight compared to infected RD-fed mice at 150 DPI. (b) *T. cruzi* infection increased the weights of the hearts compared to uninfected mice irrespective of the diets fed at 150 DPI. (c) The weights of the livers of HFD-fed mice (both uninfected and infected) were significantly increased compared to RD-fed (both uninfected and infected) mice. *T. cruzi* infection showed no significant effect on the weights of the livers. (d) Serum glucose measurements demonstrated that chronic *T. cruzi* infection has no significant effect on the levels of fasting glucose in RD-fed mice. However, the levels of fasting glucose significantly increased in infected HFD mice compared to infected RD-fed mice. (e) Infected HFD mice showed a better clearance of glucose during OGTT analysis compared to infected RD mice even though the basal levels of glucose were significantly greater in infected HFD mice compared to infected RD mice. The error bars represent standard error of the mean. ^∗^*p* ≤ 0.05, ^∗∗^*p* ≤ 0.01, or ^∗∗∗^*p* ≤ 0.001 compared to uninfected RD mice. ^#^*p* ≤ 0.05, ^##^*p* ≤ 0.01, or ^###^*p* ≤ 0.001 compared to infected RD mice. ^^^*p* ≤ 0.05, ^^^^*p* ≤ 0.01, or ^^^^^*p* ≤ 0.001 compared to uninfected HFD mice. A bar represents the mean value in (a–c).

Infection of CD1 mice with *T. cruzi* (Brazil strain) causes cardiomyopathy during the chronic stages of infection [16]. Feeding HFD during the indeterminate stage of infection led to the development of RV dilation and accelerated the development of cardiac pathology [16] (Table 1(a)). Feeding a carbohydrate-rich RD during the indeterminate stage of infection led to the development of LV dilation (Table 1(a)) [16]. While feeding different diets during the indeterminate stage of infection showed no significant difference on the survival rate of mice, we did observe significant differences in the metabolic status of animals on different diets including differences in body weight, liver weight, glucose levels, and glucose clearance at 150 DPI (Figure 1). Therefore, to understand further the effect of diet on plasma metabolites and its link to cardiomyopathy with either LV or RV dysfunction in *T. cruzi*-infected mice, we analyzed plasma metabolomic profiles of infected mice on different diets.

### 3.2. Body Weight, Glucose, and Glucose Clearance

A comparative body weight measurement analysis showed a significant decrease in the weights of infected mice compared to uninfected mice fed the same diets (either RD or HFD), as detailed below. In uninfected control groups, though the body weights of RD mice were lower compared to HFD-fed mice, there was no statistically significant difference in these groups. The body weights of infected HFD-fed mice were significantly (*p* ≤ 0.01) greater than those of infected RD-fed mice (Figure 1(a)). We also measured the weights of the hearts and livers (Figures 1(b) and 1(c)). The weights of the hearts of infected mice (RD and HFD fed) were greater than in uninfected mice fed the same respective diets (Figure 1(b)). The weights of the livers of HFD mice were significantly greater compared to RD-fed mice, irrespective of infection (Figure 1(c)). Mice showed significant differences in cholesterol levels in the hearts between uninfected and infected groups and between HFD-fed mice and RD-fed mice (Table 1(b)). The livers of HFD-fed mice (both infected and uninfected) showed significantly greater cholesterol levels than the RD-fed mice (Table 1(b)). The weights of the hearts and livers may correlate with the cardiac and hepatic levels of cholesterol, respectively, in these experimental groups (Figures 1(b) and 1(c) and Table 1(b)).

Blood glucose, after an 8-hour fast, was measured at 150 DPI (Figure 1(d)). HFD mice showed significantly higher eight-hour fasting blood glucose levels compared with RD mice in both uninfected (*p* ≤ 0.005) and infected groups (*p* ≤ 0.01). There was no significant difference in the basal glucose levels between the infected and the uninfected groups of RD-fed mice. However, the levels of glucose were significantly lower (*p* ≤ 0.05) in the infected HFD mice compared to uninfected HFD-fed mice. At 150 DPI, oral glucose tolerance was determined after an 8-hour fast (Figure 1(e)). We observed decreased glucose tolerance in the infected mice compared to the respective diet-fed uninfected mice. To our surprise, even though the basal levels of glucose were higher in uninfected HFD mice, HFD mice displayed enhanced glucose tolerance when compared to RD mice (Figure 1(d)).

### 3.3. Effect of Diet on Hepatic Lipid Metabolism during Chronic Chagas Disease

**Figure 2:**
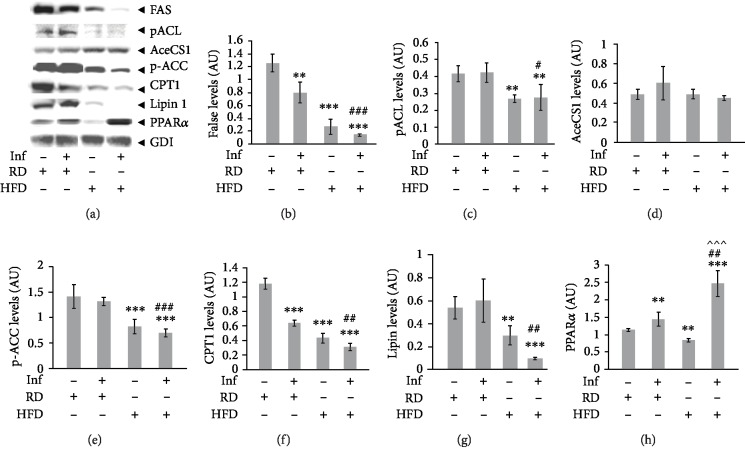
Immunoblot analysis of the livers demonstrated disrupted lipid metabolism in *T. cruzi*-infected mice and HFD further enhanced interruption of hepatic lipid metabolism during chronic Chagas disease. (a) *T. cruzi*-infected HFD-fed mice showed significantly reduced hepatic lipid metabolism compared to infected RD-fed mice as demonstrated by immunoblot analysis probed for various lipid metabolism markers such as fatty acid synthase (FAS), phosphorylated ATP-citrate lyase (pACL), phosphorylated acetyl co A carboxylase (p-ACC), carnitine palmitoyltransferase I (CPT1), and Lipin 1. Infected mice showed significantly increased hepatic levels of PPAR*α*, a regulator of fatty acid oxidation compared to uninfected mice, and HFD further enhanced the levels of PPAR*α* in the livers of infected mice. (b–h) Fold changes in the protein levels of FAS, pACL, acetyl CoA-synthase (AceCS1), pACC, CPT1, Lipin 1, and PPAR*α* were normalized to GDI expression and represented as the bar graphs (b–h, respectively). The error bars represent the standard error of the mean. ^∗^*p* ≤ 0.05, ^∗∗^*p* ≤ 0.01, or ^∗∗∗^*p* ≤ 0.001 compared to uninfected RD mice. ^#^*p* ≤ 0.05, ^##^*p* ≤ 0.01, or ^###^*p* ≤ 0.001 compared to infected RD mice. ^^^*p* ≤ 0.05, ^^^^*p* ≤ 0.01, or ^^^^^*p* ≤ 0.001 compared to uninfected HFD mice.

The livers of HFD-fed mice demonstrated higher levels of cholesterol compared to RD-fed mice suggesting an impaired lipid accumulation in HFD-fed mice (Table 1(b)). Therefore, we analyzed lipid metabolism biomarkers in the livers of RD- and HFD-fed uninfected and infected mice, and this suggested disrupted lipid metabolism in the livers of infected mice (Figure 2). Protein levels of fatty acid synthase (FAS), phosphorylated ATP-citrate lyase (pACL), acetyl CoA-synthase (AceCS1), and phosphorylated acetyl co A carboxylase (p-ACC) were determined in liver lysates of infected and uninfected mice. The levels of pACL, AceCS1, and p-ACC were not significantly altered in infected RD mice compared to uninfected RD mice; however, the protein levels of FAS, pACL, and p-ACC were significantly decreased in HFD-fed mice (both uninfected and infected groups) compared to uninfected RD mice. Immunoblot analyses of carnitine palmitoyltransferase I (CPT1), a mitochondrial enzyme that catalyzes the biosynthesis of acyl carnitines by transferring the acyl group of a long-chain fatty acyl-CoA from coenzyme A to l-carnitine [21], demonstrated significantly decreased levels of CPT1 during infection and HFD further affecting the levels (Figure 2(f)). Lipin 1, a critical regulator of intermediary fat metabolism, acts as a lipid phosphatase to dephosphorylate phosphatidic acid to form diacylglycerol—a key step in glycerolipid metabolism and triglyceride synthesis [22] was also significantly decreased in infected HFD mice compared to other groups (Figure 2(g)). Infection significantly increased hepatic PPAR*α* levels compared to uninfected RD mice, and infected HFD mice showed significantly greater levels of PPAR*α* compared to infected RD mice (Figures 2(a) and 2(h)). Although the biosynthesis of hepatic lipids was impaired during chronic *T. cruzi* infection, the levels of fatty acid oxidation increased in the livers as demonstrated by the PPAR*α* level.

### 3.4. Amino Acid Metabolism

**Figure 3:**
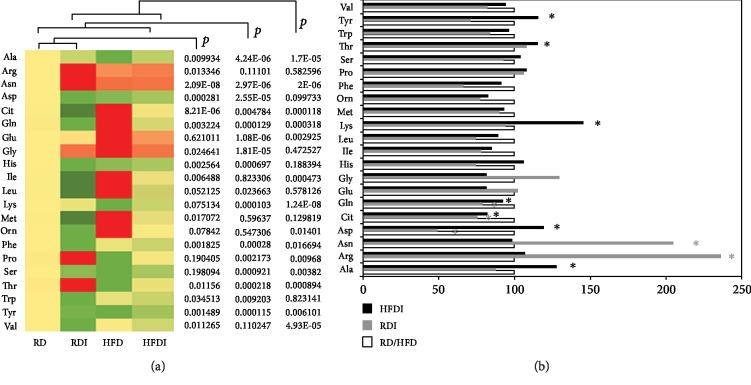
Comparison of amino acid profiling between *T. cruzi*-infected and uninfected mice fed on different diets at 150 DPI. (a) Heat map representing the serum levels of various amino acids during chronic infection in mice fed on either a RD or a HFD compared to uninfected RD mice at 150 DPI. Each row represents data for a specific metabolite, and each column represents the RD-fed uninfected (RD), RD-fed infected (RDI), HFD-fed uninfected (HFD), or HFD-fed infected (HFDI) mouse group. Different colors correspond to the different intensity level of metabolites (red > the control RD group and green < the control RD group). Statistically significant differences were tested using Student's *t*-test, and the corrected *p* values (*q*-value) are presented next to the heat map. Red, yellow, and green rectangles indicate high, moderate, and low expressions, respectively. (b) The percentage changes in the levels of amino acid metabolites in infected mice compared to the respective diet-fed (RD and HFD) uninfected mice represented by a bar graph (^∗^significant change).

A comparative amino acid profiling of serum samples demonstrated significant alterations in their levels between uninfected and infected, and between RD and HFD groups (Figure 3(a)). The levels of essential amino acids, such as isoleucine, leucine, phenylalanine, tryptophan, and valine, were decreased in both RD- and HFD-fed infected mice compared to uninfected mice fed the same respective diets (Figure 3(b)). Interestingly, the levels of lysine and threonine were significantly increased in infected HFD-fed mice compared to uninfected HFD-fed mice and infected RD-fed mice (Figure 3(b)). Both lysine and threonine are essential immune modulators [23]. Nonessential amino acid profiling also differed between infected and uninfected, and between RD and HFD diets (Figure 3(a)). Amino acids, such as alanine, aspartic acid, and tyrosine, significantly decreased in RD-fed groups and significantly increased HFD-fed groups during infection compared to their respective diet-fed uninfected groups (Figure 3(b)). The levels of asparagine and glycine significantly increased in infected RD mice compared to uninfected RD mice (Figure 3(b)). However, the levels of glycine in infected HFD mice were significantly decreased compared to uninfected HFD mice. Glutamine levels were significantly reduced during infection irrespective of diet (Figure 3(b)). We observed no significant change in the levels of glutamic acid in RD-fed mice (between uninfected and infected), but a significant decrease in infected HFD mice compared to uninfected mice. Interestingly, the serum amino acid profiling of uninfected HFD mice was significantly differed compared to uninfected RD mice (Supplemental Fig. [Supplementary-material supplementary-material-1]).

### 3.5. Biogenic Amine Profiling

**Figure 4:**
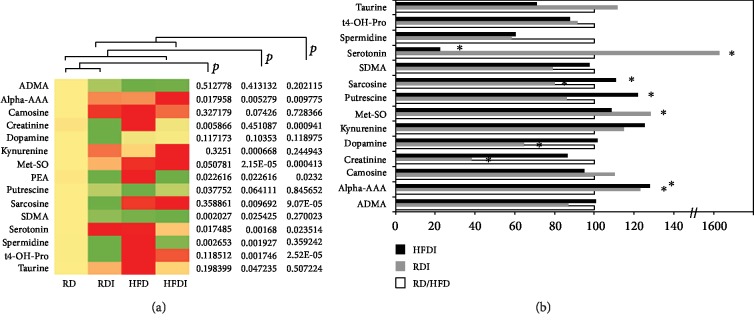
Comparison of biogenic amines profiling between *T. cruzi*-infected and uninfected mice fed on different diets at 150 DPI. (a) Heat map representing the serum levels of various biogenic amines during chronic infection in mice fed on either a RD or a HFD compared to uninfected RD mice at 150 DPI. Each row represents data for a specific metabolite, and each column represents the RD-fed uninfected (RD), RD-fed infected (RDI), HFD-fed uninfected (HFD), or HFD-fed infected (HFDI) mouse group. Different colors correspond to the different intensity level of metabolites (red > the control RD group and green < the control RD group). Statistically significant differences were tested using Student's *t*-test, and the corrected *p* values (*q*-value) are presented next to the heat map. Red, yellow, and green rectangles indicate high, moderate, and low expressions, respectively. (b) The percentage changes in the levels of biogenic amines in infected mice compared to the respective diet-fed (RD and HFD) uninfected mice represented by a bar graph (^∗^significant change).

Among the 15 bioamines analyzed, the serum levels of many bioamines measured were significantly differed between infected RD and infected HFD compared to uninfected RD mice (Figure 4). The levels of some of the bioamines measured were significantly higher in the serum of uninfected HFD mice compared to uninfected RD mice (Supplemental Fig. [Supplementary-material supplementary-material-1]) such as alpha-AAA, methionine sulfoxide (Met-SO), and serotonin. The levels of Met-SO, kynurenine, and alpha-AAA were significantly higher in the infected groups (both in HFD and RD) compared to the respective diet-fed uninfected groups (Figure 4(b)). Methionine sulfoxide is an indicator of oxidative stress. Increased kynurenine and alpha-AAA are associated with increased immune functions and metabolic dysfunctions, respectively [24]. The serum levels of serotonin significantly increased and the levels of creatinine and dopamine significantly decreased in infected RD mice compared to uninfected RD mice (Figure 4(b)), whereas the levels of sarcosine and putrescine significantly increased and serotonin significantly decreased in infected HFD mice compared to uninfected HFD mice (Figure 4(b)). However, the levels of serotonin significantly increased in infected HFD mice compared to uninfected RD mice (Figure 4(a)). It has been shown that the serum levels of serotonin increases in the patients with Takotsubo cardiomyopathy [25].

### 3.6. Lipid Metabolite Profiling

We have previously demonstrated that cardiac lipid metabolism plays a major role in the pathogenesis of cardiomyopathy in *T. cruzi*-infected mice [14, 16]. It has been shown that diets with different fat content alter cellular lipid metabolism [26]. We have demonstrated significantly reduced cardiac lipid metabolism in chronically infected HFD mice compared to infected RD mice which displayed significant RV and LV dilations, respectively. Therefore, we quantitated the levels of various lipid metabolites such as acylcarnitines, sphingolipids, and glycerol-phospholipids to analyze the effect of diet compositions in the pathogenesis of cardiomyopathy and identify potential lipid biomarkers of this disease specific to RV and LV dilations.

#### 3.6.1. Acylcarnitines

**Figure 5:**
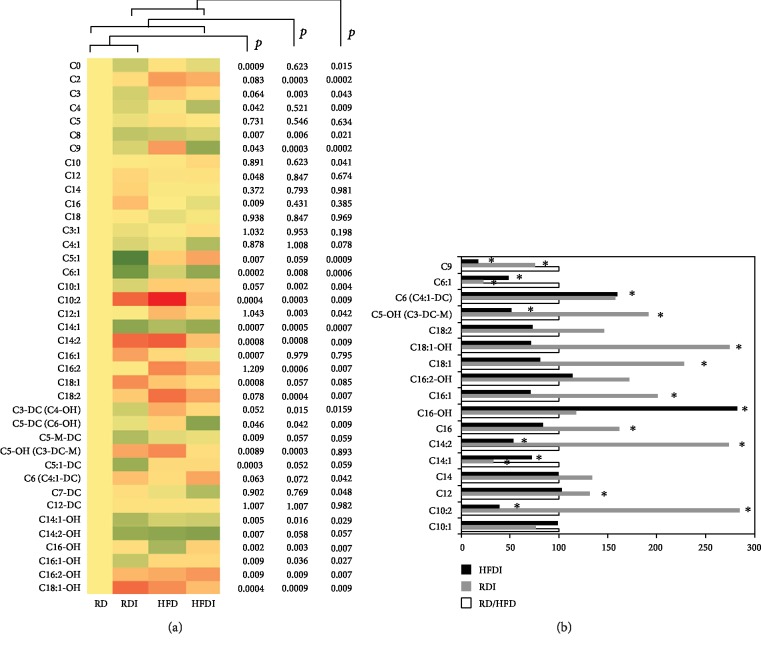
Comparison of acylcarnitine profiling between *T. cruzi*-infected and uninfected mice fed on different diets at 150 DPI. (a) Heat map representing the serum levels of various acylcarnitines during chronic infection in mice fed on either a RD or a HFD compared to uninfected RD mice at 150 DPI. Each row represents data for a specific metabolite, and each column represents the RD-fed uninfected (RD), RD-fed infected (RDI), HFD-fed uninfected (HFD), or HFD-fed infected (HFDI) mouse group. Different colors correspond to the different intensity level of metabolites (red > the control RD group and green < the control RD group). Statistically significant differences were tested using Student's *t*-test, and the corrected *p* values (*q*-value) are presented next to the heat map. Red, yellow, and green rectangles indicate high, moderate, and low expressions, respectively. (b) The percentage changes in the levels of acylcarnitines in infected mice compared to the respective diet-fed (RD and HFD) uninfected mice represented by a bar graph (^∗^significant change).

Most of the measured acylcarnitines were significantly altered between uninfected and infected groups and between different diet groups (Figure 5, Supplemental Fig. [Supplementary-material supplementary-material-1]). The serum levels of C2, C3, C14, and C18 were significantly altered between uninfected RD and uninfected HFD groups (Supplemental Fig. [Supplementary-material supplementary-material-1]). The serum levels of carnitine (C0) significantly reduced during infection compared to uninfected groups fed the same respective diets. Among the analyzed carnitines, the levels of C0, C14:1, C14:1-OH, C14:2-OH, C4, C4:1, C5-DC (C6-OH), C5-M-DC, C6:1, C8, and C9 were significantly lowered in the infected mice (RD and HFD mice) compared to uninfected RD mice (Figure 5(a)). However, the levels of some of the carnitines, such as C10:2, C12, C14, C14:2, C16, C16:1, C18:1, C18:1-OH, and C5-OH (C3-DC-M), were significantly increased in infected RD mice compared to infected HFD mice and uninfected RD controls (Figure 5(a)). The serum levels of C10, C10:1, C12:1, C16-OH, C16:1-OH, C16:2, C16:2-OH, C18:2, C2, C3, C3-DC (C4-OH), C3:1, C5:1, C5:1-DC, and C6 (C4:1-DC) significantly increased in infected HFD mice compared to infected RD mice and uninfected RD controls (Figure 5(a)).

The serum levels of C9, C6:1, and C14:1 were significantly reduced, and the levels of C6 (C4:1-DC) significantly increased in infected RD and infected HFD mice compared to the respective diet-fed uninfected groups (Figure 5(b)) and thus may represent specific biomarkers of CCM. The levels of C5-OH (C3-DC-M), C18:1-OH, C18:1, C16:1, C16, C14, and C12 were significantly higher in infected RD mice compared uninfected RD mice as well as HFD (both uninfected and infected) mice, which may represent as specific biomarkers of LV enlargement in CCM pathogenesis (Figures 5(a) and 5(b) and supplemental Fig. [Supplementary-material supplementary-material-1]). And increased serum levels of C16: OH may represent as a biomarker of RV dilation in CCM pathogenesis.

#### 3.6.2. Sphingolipids

**Figure 6:**
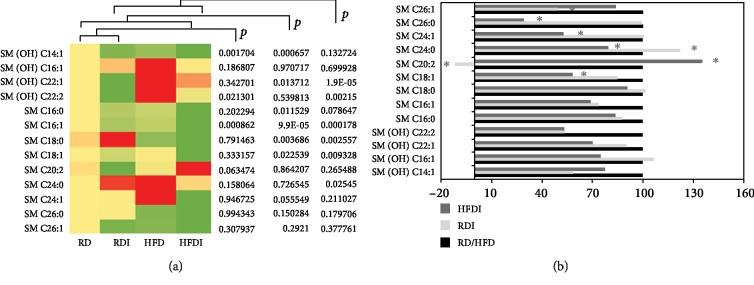
Comparison of sphingolipid profiling between *T. cruzi*-infected and uninfected mice fed on different diets at 150 DPI. (a) Heat map representing the serum levels of various sphingolipids during chronic infection in mice fed on either a RD or a HFD compared to uninfected RD mice at 135 DPI. Each row represents data for a specific metabolite, and each column represents the RD-fed uninfected (RD), RD-fed infected (RDI), HFD-fed uninfected (HFD), or HFD-fed infected (HFDI) mouse group. Different colors correspond to the different intensity level of metabolites (red > the control RD group and green < the control RD group). Statistically significant differences were tested using Student's *t*-test, and the corrected *p* values (*q*-value) are presented next to the heat map. Red, yellow, and green rectangles indicate high, moderate, and low expressions, respectively. (b) The percentage changes in the levels of sphingolipid metabolites in infected mice compared to the respective diet-fed (RD and HFD) uninfected mice represented by a bar graph (^∗^significant change).

We quantified 14 sphingolipids, including both hydroxylated (*n* = 5) and nonhydroxylated (*n* = 9) ceramide phosphocholines (sphingomyelins) (Figure 6(a)). HFD significantly increased the serum levels of SM (OH) C22:1, C22:2, C24:0, and C24:1 compared to RD (Supplemental Fig. [Supplementary-material supplementary-material-1]) in uninfected mice. In general, the serum levels of other sphingomyelins were reduced in HFD-fed (+/- infection) mice compared to RD controls, except the levels of SM C24:0 and SM C24:1, which significantly increased in HFD-fed controls (Figure 6(a)).

The levels of many sphingolipids such as SM C18:1, C16:1, and C16, and SM (OH) C22:2, C22:1, and C14:1 significantly decreased during infection in mice compared to their respective diet-fed control groups (Figure 6(b)). In addition, SM C 20:2 significantly decreased in infected RD mice and significantly increased in infected HFD mice compared to other groups (Figure 6(b)). These data suggest that the serum levels of SM C 20:2 can be a potential biomarker of CCM pathogenesis.

#### 3.6.3. Glycerophospholipids

**Figure 7:**
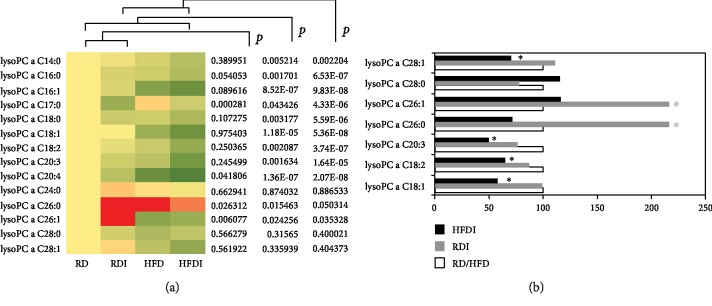
Comparison of 14 lysophosphatidylcholines profiling between *T. cruzi*-infected and uninfected mice fed on different diets at 150 DPI. (a) Heat map representing the serum levels of various lysophosphatidylcholines during chronic infection in mice fed on either a RD or a HFD compared to uninfected RD mice at 150 DPI. Each row represents data for a specific metabolite, and each column represents the RD-fed uninfected (RD), RD-fed infected (RDI), HFD-fed uninfected (HFD), or HFD-fed infected (HFDI) mouse group. Different colors correspond to the different intensity level of metabolites (red > the control RD group and green < the control RD group). Statistically significant differences were tested using Student's *t*-test, and the corrected *p* values (*q*-value) are presented next to the heat map. Red, yellow, and green rectangles indicate high, moderate, and low expressions, respectively. (b) The percentage changes in the levels of LPC metabolites in infected mice compared to the respective diet-fed (RD and HFD) uninfected mice represented by a bar graph (^∗^significant change).

A total of 87 glycerophospholipids (*n* = 14 lysophosphatidylcholines; *n* = 73 phosphocholines) were quantified in the collected serum samples (Figure 7). Of 14 quantified lysophosphatidylcholines, 8 metabolites (57%) such as lysoPC as' C14:0, C16:0, C16:1, C18:0, C18:1, C18:2, C20:3, and C20:4 were significantly decreased in uninfected HFD-fed mice (Supplemental [Supplementary-material supplementary-material-1]) compared to uninfected RD mice. *T. cruzi* infection significantly reduced the levels of a lysoPC C17:0 in both RD- and HFD-fed mice compared to their respective diet-fed uninfected groups (Figure 7(b)). The levels of lysoPCs C26:0 and C26:1 significantly increased in infected RD groups compared to uninfected RD groups. Although the levels of many lysoPCs significantly decreased in HFD-fed groups compared to RD-fed groups (Figure 7(a), Supplemental Fig. [Supplementary-material supplementary-material-1]), the levels of lysoPCs C18:1, C18:2, C20:3, and C28:1 significantly decreased in infected HFD groups compared to uninfected HFD groups (Figure 7(b)), which could be potential markers of RV dilation in *T. cruzi*-infected mice (Figure 7(b)).

**Figure 8:**
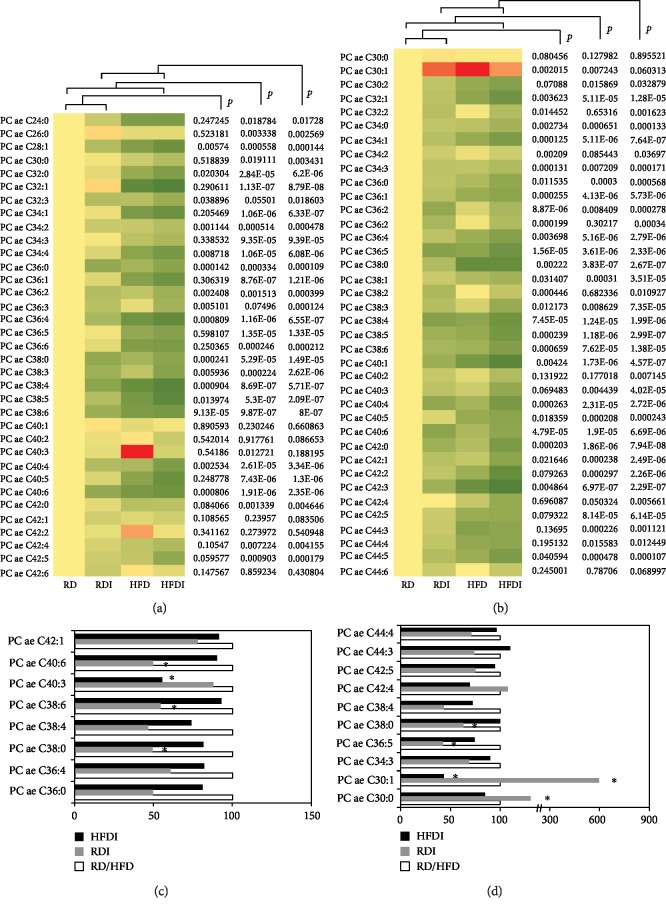
Comparison of phosphocholine (73) profiling between *T. cruzi*-infected and uninfected mice fed on different diets at 150 DPI. (a, b) Heat map representing the serum levels of various glycerol-phospholipids that included (a) diacyl PC and (b) acyl-alkyl PC during chronic infection in mice fed on either a RD or a HFD compared to uninfected RD mice at 150 DPI. Each row represents data for a specific metabolite, and each column represents the RD-fed uninfected (RD), RD-fed infected (RDI), HFD-fed uninfected (HFD), or HFD-fed infected (HFDI) mouse group. Different colors correspond to the different intensity level of metabolites (red > the control RD group and green < the control RD group). Statistically significant differences were tested using Student's *t*-test, and the corrected *p* values (*q*-value) are presented next to the heat map. Red, yellow, and green rectangles indicate high, moderate, and low expressions, respectively. (c, d) The percentage changes in the levels of phosphocholine metabolites that included (c) diacyl PC and (d) acyl-alkyl PC in infected mice compared to the respective diet-fed (RD and HFD) uninfected mice represented by a bar graph (^∗^significant change).

HFD significantly decreased the levels of diacyl and acyl-alkyl phosphatidylcholines (Supplemental [Supplementary-material supplementary-material-1] and b, respectively) compared to RD fed in uninfected groups. 26 of the 34 quantified diacyl phosphatidylcholines (PC-aa) were significantly reduced in HFD groups compared to RD groups (Figure 8(a) and Supplemental Fig. [Supplementary-material supplementary-material-1]). Between the uninfected and the infected groups, 16 PC-aa's significantly decreased in infected groups compared to uninfected controls, and HFD further reduced the levels of these PC-aa's (Figure 8(a)). The levels of PC-aa C36:3, PC-aa-C38, and PC-aa-C38:3 were significantly decreased in infected RD and HFD mice compared to uninfected (both RD and HFD) mice (Figures 8(a) and 8(c)). We observed a similar trend in the levels of acyl-alkyl phosphatidylcholines (PC-ae), most of which were reduced in infected groups and even further reduced in HFD-infected mice (Figure 8(b) and Supplemental Fig. [Supplementary-material supplementary-material-1]). The levels of PC-ae-C36:2, PC-ae-C38:3, PC-ae-C38:4, PC-ae-C40:4, PC-ae-C40:6, and PC-ae-C42 were significantly reduced in infected mice compared to uninfected RD mice (Figure 8(b)). However, the levels of PC-ae C30:1 was significantly higher in the infected groups (both in RD and HFD fed) compared to uninfected RD mice (Figure 8(b)). The levels of PC-ae-C38:2 significantly reduced compared to all the other groups and thus may represent as a biomarker of LV-specific dilation in CCM.

## 4. Discussion

CCM is the most important clinical manifestation of Chagas disease, resulting in the mortality and morbidity in the endemic regions of Latin America. The factors responsible for the transition between asymptomatic to symptomatic CCM forms are not completely understood, and thus, the prognosis of chronic Chagas disease is difficult. Moreover, Chagas patients display various severity cardiac forms during the chronic stages [27], and the severity-specific markers are not greatly explored that could have served as a set of biomarkers in the prognosis of CCM pathogenesis. Using murine models of Chagas disease, we previously demonstrated that feeding a high calorie fat or carbohydrate diets may differentially influence the cardiac pathology and outcome of chronic Chagas disease [16]. *T. cruzi*-infected mice fed RD developed LV dilation and mice fed HFD developed RV dilation at 160 DPI [16]. Histological analysis demonstrated increased lipid droplets and enlarged capillaries in the RVs of infected HFD mice compared to infected RD mice [16]. We demonstrated that cholesterol efflux mechanisms, lipid oxidation, and mitochondrial dysfunction were all affected by HFD during chronic stages of infection, resulting in aggravated cardiac lipotoxicity, vascular and cardiac accumulation of lipid droplets, and vascular dilation [16]. In the later stages of chronic infection, HFD-fed infected mice showed lower levels of proinflammatory markers (TNF*α* and IFN*γ*) in the hearts compared to RD-fed infected mice although the levels of inflammatory cells were higher in infected HFD mice [16]. However, the levels of proinflammatory cytokines were significantly increased in infected RD mice compared to uninfected RD-fed mice suggesting that inflammation may play a major role in LV enlargement. Diets regulate the immunometabolic status of the host, which influences the severity of the disease, and thus, the serum metabolic profiling differs between different pathological conditions. Changes in host metabolomic profiling were demonstrated during acute stages of infection in a murine model of *T. cruzi* infection [19]; however, those observations cannot be extrapolated to evaluate the metabolic changes during the chronic stage of infection (occurring after several weeks in the murine model and after years/decades of infection in human disease), and the disease pathways are different between the acute and the chronic stage of infection. The current study analyzed the serum metabolomic profile of *T. cruzi*-infected mice fed on different diets that specifically developed RV and LV dilations. These metabolomic data demonstrate significant differences in the serum levels of amino acids, biogenic amines, and lipid metabolites between chronic *T. cruzi*-infected and uninfected mice, which further differed between mice fed fat-rich (HFD) and carbohydrate-rich (RD) diets. HFD and RD also differentially regulated hepatic and systemic lipid and glucose metabolism, which could influence cardiac morphology, physiology, and pathology during chronic *T. cruzi* infection. These metabolomic studies provide new insights into metabolic changes that could significantly influence cardiomyopathy during chronic Chagas disease and may define new biomarkers to identify Chagas patients at risk of cardiomyopathy.

Infected mice fed on HFD showed significantly greater body weight gain compared to infected RD mice, while no significant difference was observed between the HFD-fed and the RD-fed uninfected mice consistent with a gain in body weight with age with both diets. It is likely that the amount of carbohydrates (70% kcal) in the RD might have allowed uninfected RD-fed mice to gain a similar amount of weight as uninfected HFD-fed mice. In infected animals, however, HFD-fed mice had increased body weights that may be a consequence of enlarged livers (Figure 1; no significant change was observed with other organs, data not provided). HFD may further aggravate hepatic dysfunction during chronic *T. cruzi* infection and is reflected in the difference in serum metabolomic profiles between infected mice on HFD or RD. The basal glucose levels were significantly higher in HFD-fed groups compared to RD-fed groups (both infected and uninfected), suggesting that there is a difference in metabolic requirements between HFD- and RD-fed mice. HFD fed may prefer to utilize mainly lipids (fatty acids) as their energy source compared to glucose since excessive fat is available in their diet, especially in the infected HFD mice as indicated by significantly increased PPAR*α* levels (Figure 2). However, infected mice (both RD and HFD mice) had a decreased glucose tolerance compared to uninfected mice, which agrees with our previous report that *T. cruzi* infection causes pancreatitis and deregulated insulin signaling that persists into the stage of chronic infection [14].

Increased uptake of dietary lipids in HFD groups (both uninfected and infected) might have caused reduction in lipid biosynthesis metabolism as demonstrated by significantly decreased levels of lipid metabolism biomarkers compared to RD-fed mice (Figure 2). Fatty acid anabolism was significantly decreased, as shown by reduced protein levels of fatty acid synthase (FAS), which is involved in the biosynthesis of long-chain fatty acids from acetyl CoA and malonyl CoA (Figures 2(a) and 2(b)) [28]. However, the initial steps of fatty acid biosynthesis were not significantly altered during infection, as demonstrated by the protein levels of phosphorylated ATP-citrate lyase (pACL), acetyl CoA-synthase (AceCS1), and phosphorylated acetyl co A carboxylase (p-ACC) in the livers of RD-fed mice compared to uninfected RD-fed mice (Figures 2(a), 2(c), 2(d), and 2(e)). pACL catalyzes the synthesis of acetyl CoA and oxaloacetate [29]; the levels of which are unaltered in the livers during infection in RD-fed mice (Figures 2(a) and 2(c)). The formation of acetyl CoA and oxaloacetate in the cytoplasm is the key step for the biosynthesis of fatty acids, cholesterol, and acetylcholine [26]. In fact, the levels of AceCS1, an enzyme that synthesizes acetyl CoA in the cytoplasm, a precursor for fatty acid and lipid biosynthesis, slightly increased (not significantly) in the infected RD mice compared to uninfected RD mice [30]. However, the levels of pACL and p-ACC significantly decreased in HFD (uninfected and infected) mice compared to RD (uninfected and infected) mice (Figures 2(a), 2(c), and (e)). A significant decrease in the levels of pACL suggests that acetyl CoA synthesis is impaired or reduced in the livers of HFD-fed mice, which could impact lipid biosynthesis. Interestingly, the levels of PPAR*α* were significantly increased in infected groups suggesting a high demand for fatty acids during chronic *T. cruzi* infection. Increased fatty acid oxidation in the livers could cause lipotoxicity and impair liver functions (carbohydrate, protein, and fat metabolism) resulting in distinct serum metabolomic profiles.

The levels of amino acids significantly differed between infected and uninfected, and between RD- and HFD-fed groups. The levels of many essential and nonessential amino acids were significantly reduced in infected mice compared to uninfected groups. It is well known that amino acids play a major role in regulating the immune system, nutritional status, and energy homeostasis [23, 31]. Leucine, isoleucine, and valine are the branched chain aa's that influences immune responses which were significantly decreased in infected mice. Glutamine, which regulates B cell differentiation to plasma cells and proliferation of T cells, is significantly reduced in infected mice compared to uninfected groups (Figure 3) [32]. Reduced glutamine levels and metabolism impacts the levels of its end product citrulline in infected mice, especially in infected RD mice which is significantly decreased compared to infected HFD mice (Figure 3). Increased glycine levels in HFD mice (uninfected) are associated with regional body fat and altered energy homeostasis compared to uninfected RD-fed mice [33]. However, the levels of glycine were significantly increased in infected RD mice and significantly decreased in infected HFD mice compared to the respective uninfected groups (Figure 3); this suggests that the composition of a diet could significantly alter hepatic amino acid metabolism and energy homeostasis during chronic *T. cruzi* infection. Based on our data, we hypothesize that significantly decreased serum levels of citrulline and glutamine are associated with CCM pathogenesis and increased arginine and asparagine levels are associated with LV dysfunction and dilation (Table 2).

To facilitate the required energy supply, the human body oxidizes significant amounts of lipids besides glucose [9]. We demonstrated significantly increased PPAR*α*, a regulatory of fatty acid oxidation in the livers of infected mice especially in infected HFD mice (Figure 2). L-carnitine transports activated long-chain fatty acids from the cytosol into the mitochondrion, a step crucial for fatty acid oxidation [21]. Defects in fatty acid oxidation result in increased plasma acyl carnitine levels, which are well supported by the acyl carnitine profiles of patients with a fatty acid oxidation defect [34]. Incomplete fatty acid oxidation may cause increased plasma acyl carnitine levels [35]. Our data showing significantly increased levels of serum carnitines especially long- and medium-chain acyl carnitines can be attributed to low fatty acid oxidation rates due to decreased levels of hepatic CPT1 (even with increased PPAR*α* levels) during chronic infection compared to uninfected RD groups (Figure 2(a)). The branched chain amino acid-derived C3, C4, and C5 carnitines were significantly lower in infected RD mice compared to uninfected RD mice and infected HFD mice (except C4 carnitine). In general, the serum levels of long- and medium-chain acyl carnitines were significantly greater in uninfected HFD mice compared to uninfected RD mice (supplemental Fig. [Supplementary-material supplementary-material-1]). Compared to their respective diet-fed uninfected groups, infected groups displayed a significantly different pattern of serum acyl carnitine profile between infected RD and HFD mice. In support of these data, we found decreased levels of C9, C6:1, and C14:1 and increased levels of C6 (C4:1-DC) as the impaired carnitine metabolism markers in CCM pathogenesis (Table 2). Based on these data, we propose that increased levels of C16:OH and decreased serum levels of C16 and C5-OH (C3-DC-M) and increased levels of C12, C16, C16:1, C18:1, C18:1OH, and C5-OH (C3-DC-M) may be the distinct markers of RV and LV dilations, respectively, in CCM (Table 2).

LPC is an important signaling molecule with diverse biological functions, which is involved in regulating cellular proliferation, tumor cell invasion, and inflammation [36]. In particular, LPC is a chemotactic factor that stimulates immune cells and regulates the balance between pro- and anti-inflammatory cytokines [37]. As chronic Chagas cardiomyopathy is associated with proinflammatory signals [27, 38], one might expect LPCs to be increased in association with cardiac pathology. However, our results demonstrated that decreased levels of serum LPCs are associated with the pathogenesis of cardiomyopathy during chronic infection. The levels of lysoPCs C18:1, C18:2, C20:3, and C28:1, significantly decreased in infected HFD groups compared to all other groups (Figure 7(b)), which could be potential markers of RV dilation *in T. cruzi*-infected mice (Figure 7(b)) (Table 2). Infected mice showed significantly decreased levels of LPCs and other glycerophospholipids (diacyl-PC and acyl-alkyl-PC) compared to uninfected RD-fed mice. Feeding a HFD during *T. cruzi* chronic infection further decreased the levels of glycerophospholipids compared to infected RD mice and increased susceptibility to develop cardiomyopathy. Our previous data demonstrated that HFD decreased inflammatory signaling in the hearts of chronically infected mice, which may be attributed to the low levels of LPCs.

We have previously reported on the accumulation of lipids in different organs, including the liver and the heart, in murine Chagas disease [9, 39]. We also showed significantly decreased hepatic neutral lipids, increased hepatic cholesterol levels, and decreased fatty acid synthesis and mitochondrial oxidation in a murine model of acute *T. cruzi* infection [39]. In the present study, our results suggest that even during the chronic stages of infection, hepatic fatty acid and cholesterol metabolism remain significantly altered, especially in the mice fed on a HFD (Figure 3). These alterations could affect cardiac lipid metabolism. In support of this hypothesis, we have found that cardiac lipid metabolism was significantly reduced in infected HFD mice that developed RV dilation compared to infected RD mice during the chronic stage of infection [16].

## 5. Conclusion

CCM is an important disease in the endemic regions of Latin America. The majority of *T. cruzi*-infected patients are asymptomatic until they present with severe and typically irreversible cardiac complications. Though multiple biomarkers have been identified in Chagas patients, they are not specific to disease severity nor are they predictors of progression in asymptomatic patients. The identification of new metabolic biomarkers would greatly facilitate studies on disease progression and prevention and allow the identification of the subgroup of patients at a higher risk to develop ventricular-specific dilation and heart failure. Our data demonstrates many metabolic alterations in the experimental chronic Chagas disease model, which can be attributed to diet composition. Importantly, HFD induced much greater variations in serum metabolite levels compared to RD during chronic infection, which may impair liver functions by increased loads of lipids, which may in turn affect cardiac functions. These data help link the risk of developing cardiomyopathy to the levels of these serum metabolites (Table 2). Serum metabolite analysis in patients with indeterminate Chagas disease may, therefore, help identify those at risk for developing cardiomyopathy, providing a much needed early tool to prevent the progression of cardiomyopathy and may be useful in gauging cardiac dysfunction. Investigations of serum metabolomic profiles in various stages of human Chagas disease hence warrants further analyses.

## Figures and Tables

**(a):** 

	Composition (%kcal)		LVID (mm)	RVID (mm)
Carb-rich diet (RD) (research diet #D12450J)	Carbohydrate -70 Fat -10 Protein -20	Uninfected Infected	3.5 ± 0.08 4.5±0.2^∗∗∗^	2.1 ± 0.05 1.98 ± 0.1
High fat diet (HFD) (research diet #D12492)	Carbohydrate -20 Fat -60 Protein -20	Uninfected Infected	3.5 ± 0.08 4.5±0.2^∗∗∗^	1.97 ± 0.09 2.9±0.12^∗∗∗^^##^^^

**(b):** 

		RD	HFD
Heart	Uninfected	2.64 ± 0.26	3.843±0.39^∗∗^
	Infected	3.158±0.18^∗∗^	3.77±0.32^∗∗^^/#^
Liver	Uninfected	3.867 ± 0.81	7.547±0.52^∗∗∗^
	Infected	3.68 ± 0.62	6.587±0.49^∗∗∗^^###^

**Table 2:** A list of predicted metabolic biomarkers involved in the pathogenesis of CCM and RV and LV dilations in the murine models of chronic Chagas disease (arrows indicate decrease or increase).

Amino acids	CCM	RV	LV
	Citrulline↓ Glutamine↓		Arginine↑ Asparagine↑
Amino derivative			
	Alpha-AAA↑ PEA↓ Spermidine↓	Sarcosine↑	Sarcosine↓
Carnitines			
	C9↓ C6:1↓ C14:1↓ C6 (C4:1-DC)↑	C16:OH↑ C16↓ C5-OH (C3-DC-M)↓	C12↑ C16↑ C16:1↑ C18:1↑ C18:1OH↑ C5-OH (C3-DC-M)↑
Sphingolipids			
	SM(OH)C14:1↓ SM C16:1↓	SM C20:2↑	SM C20:2↓
lysoPC			
		lysoPC a C18:1↓ lysoPC a C18:2↓ lysoPC a C20:3↓ lysoPC a C28:1↓	lysoPC a C26:0↑ lysoPC a C26:1↑
PC-aa/ae			
	PC-aa-C36:3↓ PC-aa-C38↓ PC-aa-C38:3↓ PC-ae-C36:2↓ PC-ae-C38:3↓ PC-ae-C38:4↓ PC-ae-C40:4↓ PC-ae-C40:6↓ PC-ae-C42↓		PC-ae-C38:2↓

## Data Availability

The metabolomic data used to support the findings of this study are included within the article.

## Supplementary Materials

Supplementary Materials The representative bar graphs showing the difference in the serum levels of (1) amino acids and amines and (2 and 3) lipid metabolites between uninfected mice fed HFD and RD.
