# Supplementary figures and images for: Diet Alters Serum Metabolomic Profiling in the Mouse Model of Chronic Chagas Cardiomyopathy
Source: Dis Markers. 2019 Dec 20;2019:4956016. doi: 10.1155/2019/4956016 (PMC6948343; doi:10.1155/2019/4956016)

Supplemental Fig. 1

**a**

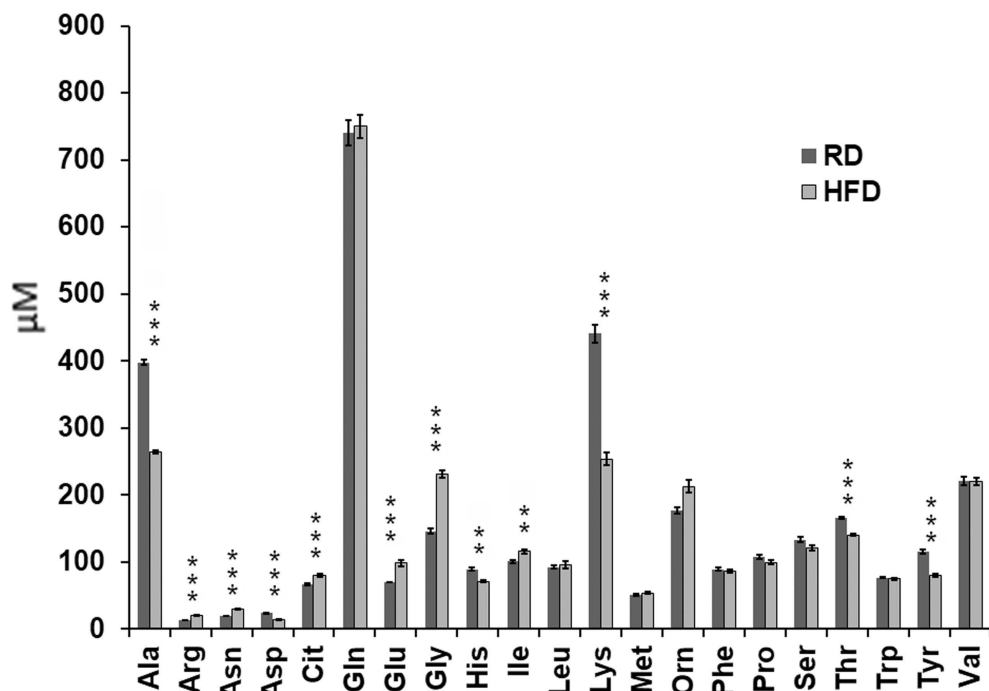

**b**

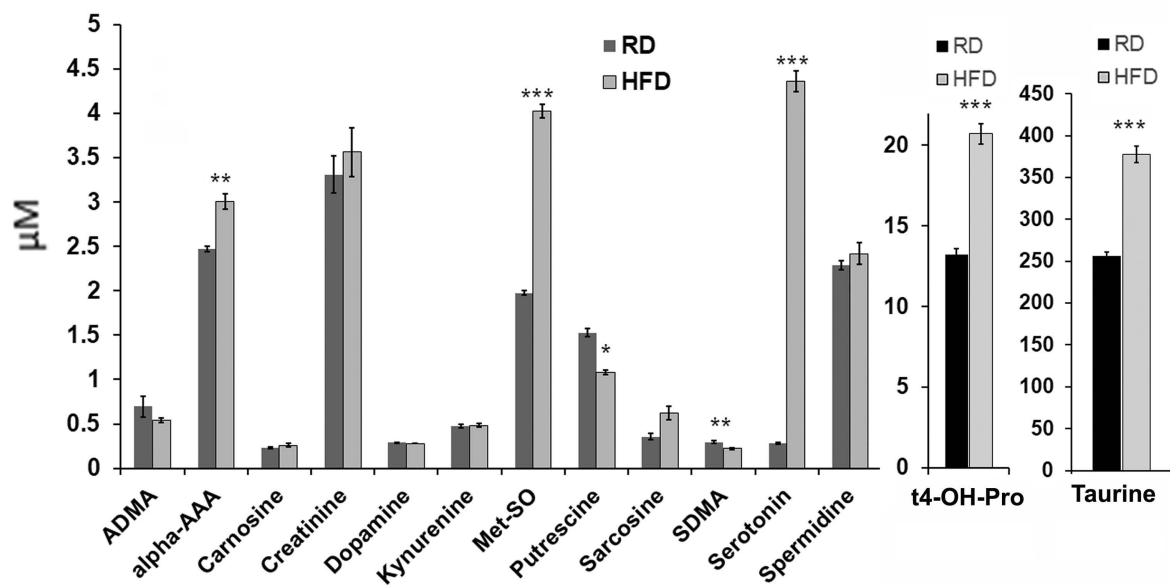

Supplemental Fig. 3

a

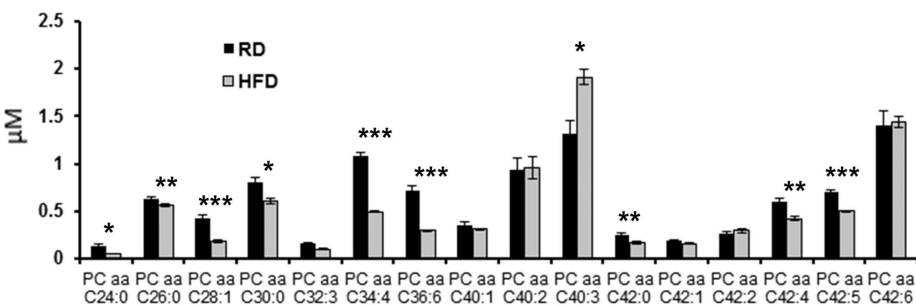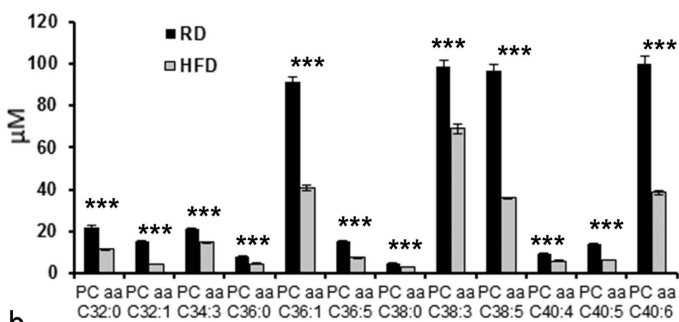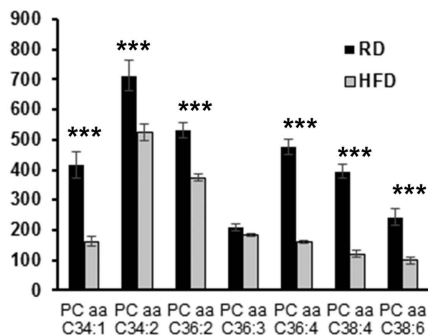

b

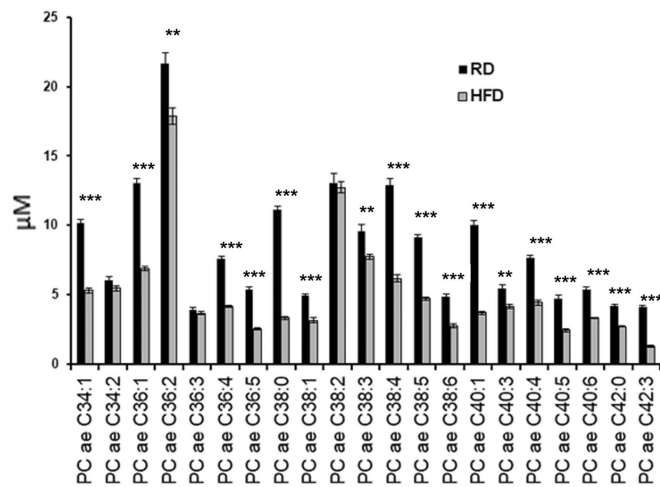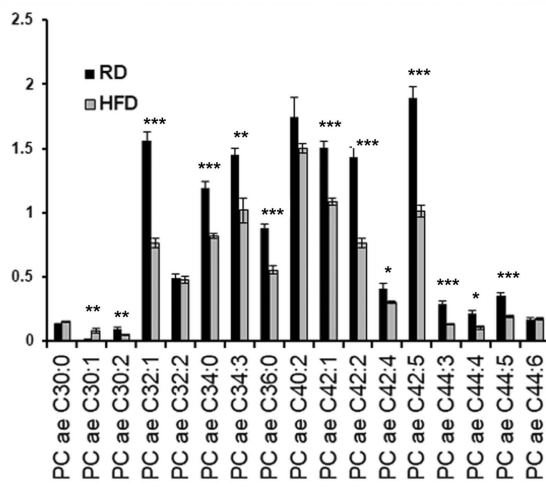

# Supplemental Fig. 2

a

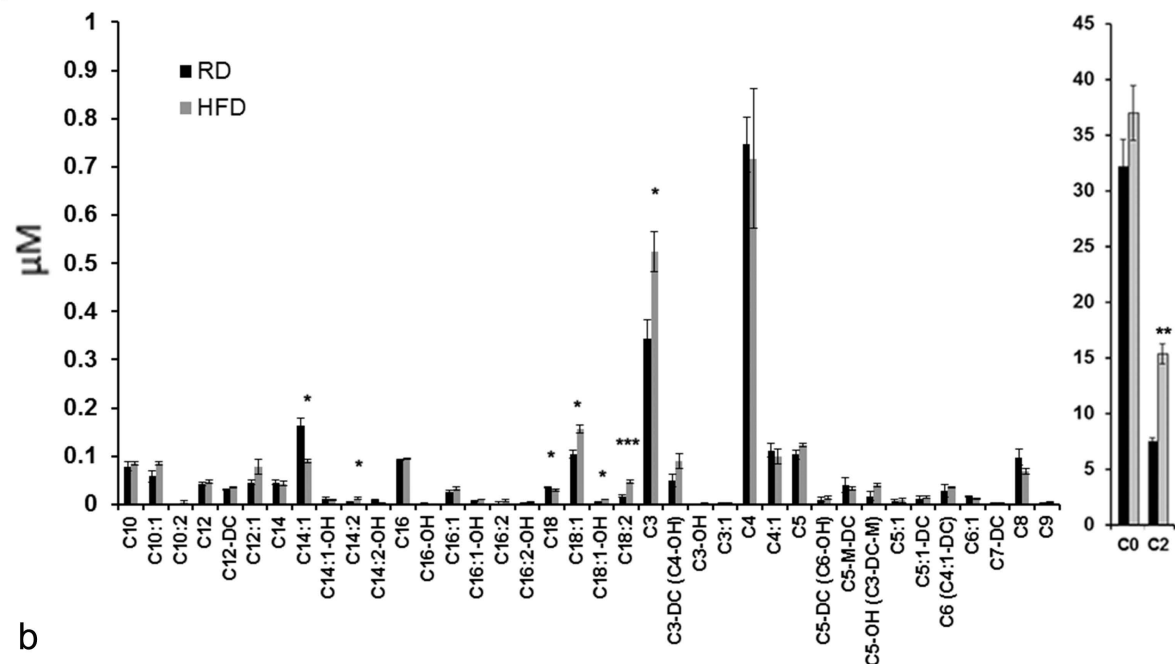

b

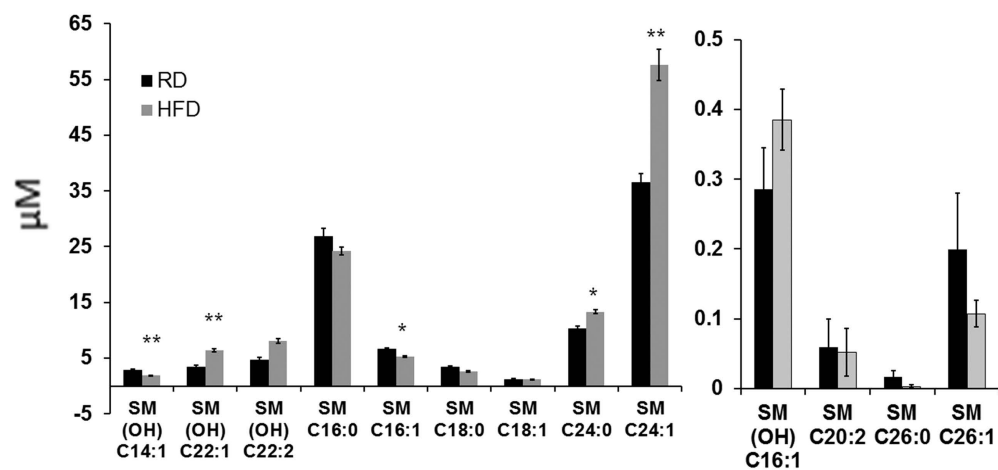

c

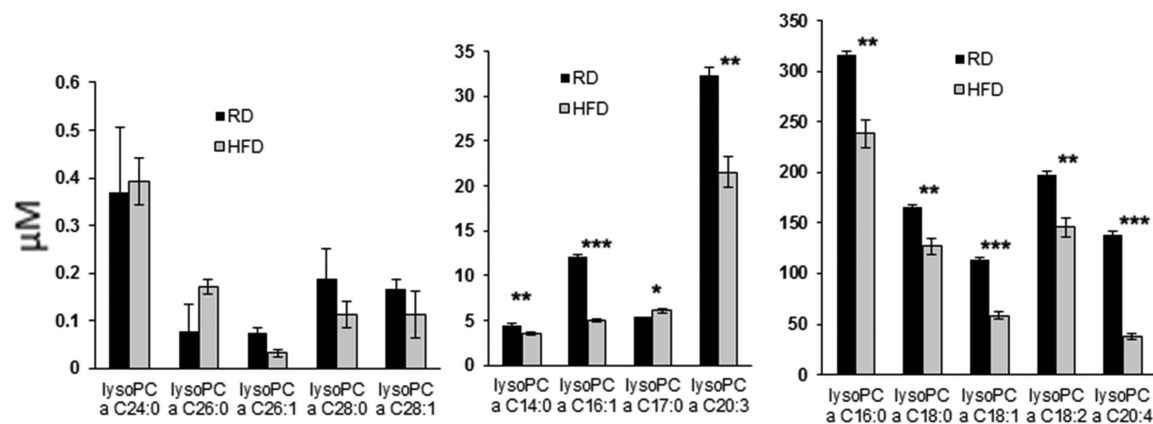

Supplement: Supplementary Materials — The representative bar graphs showing the difference in the serum levels of (1) amino acids and amines and (2 and 3) lipid metabolites between uninfected mice fed HFD and RD. [file 4956016.f1.pdf]
